# Urinary Diseases

**Published:** 1840-01

**Authors:** 


*Urinary Diseases.—Proximate Cause of Diabetes.*

Derangement of the digestive functions is here unquestionably the head and front of the offence. This derangement too is of a very peculiar kind: the stomach has a disposition to fabricate sugar to a preternatural extent out of the food, particularly the vegetable food, taken into it; for the disease is functional in every part, and is actually *disease* only because the *degree* of a natural process is surpassed—the healthy stomach generates sugar to a limited extent; the stomach of the melituric individual generates sugar in excess. The sugar produced in health is without doubt decomposed in the after-stages of digestion; it does not appear in any of the fluids of the body, nor in any of the secretions save milk, of which it is an element, nor in the feces; in the melituric state the sugar engendered is not decomposed; it enters the circulation. To explain all the rest of the disease,—the fever, and wasting, and excessive secretion of urine, &c., becomes easy after this: there is a substance mingled with the blood which is foreign to its constitution, and which consequently poisons life in its fountain head. The derangement of the kidneys is purely secondary, and is consequent upon its efforts as grand purifier of the system along with the lung, to free the circulating fluid of the cause of all the evil—the sugar. The morbid changes discovered in the kidneys, after death from melituria, have even generally been extremely trifling, they have mostly been such as indicated simple increase of functional activity; and this, in fact, is all that the kidneys can be rightfully charged with,—if their efforts are not much rather beneficent than prejudicial; for my own persuasion is, that without energetic action on their part, the disease would prove far more rapidly fatal than it does.

*Treatment.*— Dr. Willis remarks—"*Could we discover any means of preventing the stomach from forming sugar, we*

*should, I believe, succeed in curing the disease.* To this end the efforts of practitioners ought in future, as I imagine, to be directed.

He reprobates large doses of opium, but would employ the remedy in doses of one or two grains.

He naturally lauds the sudatorium. It happens, he says, very rarely indeed that a melituric patient is found who resists this powerful agent, which cause large drops of sweat to start upon the skin where no trace of moisture had been seen for months. The sudatorium has even been held as a complete prophylactic of the disease we are discussing: and it is curious to learn that in Russia, as Dr. Lefevre informs us, where every peasant and artizan makes use of the sudatorium, the disease is unknown. He had himself looked in vain through the records of the principal civil and military hospitals of the Russian empire, for the mention of a single case; and Sir James Wylie had not met with it once among upwards of two millions of soldiers who had passed under his notice as general military inspector. But the sudatorium, though seemingly useful, ultimately failed in two cases of Dr. Watson's. Still it is a valuable adjuct to our remedies.

He seems to anticipate much from magnesia.

*Proposal to exclude Insalivation.*—Dr. Willis, in his laudable anxiety to seize on any remedy for so desperate a disorder, as drowning men are known to catch at straws, proposes to exclude, so far as may be, the action of saliva from the process of digestion. He does this, because the experiments of Leuchs, Schwann, and Mueller have shewn the power possessed by saliva of converting starch into sugar.

Would any thing be gained by administering food to patients affected with melituria, without suffering it to undergo the ordinary preliminary mastication, thereby preventing it from being mingled with saliva? Would food that has not been mixed with saliva, thrive with man for any length of time? I believe it would.

Could vegetable food be administered to melituric patients without prejudice through an œsophagus tube? If the agent in the conversion of fecula into sugar be saliva, as seems certain from the experiments of the distinguished physiologists referred to, we should expect to avoid this conversion by preventing any saliva from reaching the stomach. Chemistry, which has served medicine so efficiently and on so many occasions, ought surely to come to her aid in this instance, and show as well what will *prevent* as what will *cause* the formation of sugar from starch.

*Are Diabetic Patients Intellectual?*—It has been remarked, says Dr. Willis, that those who suffer from diabetes are generally individuals of superior intelligence. Hufeland, writing after he had passed something like sixty years in the exercise of his profession, says he never met with a *stupid* man affected with diabetes. From what we have seen of diabetes, it has appeared to us that individuals affected with it have been much like the ordinary run of mortals, some clever and some obtuse. Of this we are certain, that one of the *stupidest* of clod-hoppers died of it under our observation. Hufeland's, we should conceive to be one of those whimsical Continental crotchets, that make plain folks on this side of the water stare.

*Mode of giving Alkalies for Lithic Acid Gravel.*—Dr. Willis, after considering the caustic alkalies as nauseous, if not acrimonious, (a censure which is perhaps too harsh and indiscriminating,) and approving, of course, of the bicarbonates of potash and soda, observes that it is of great importance to exhibit them in a state of very plentiful dilution. Equal quantities, he says, of some of the natural mineral waters that actually contain but one or less than one part of bicarbonate of soda in 200 of the fluid, are more potent in rendering the urine alkaline than a solution of the same salt in the ratio of one to fifty or sixty. A dram, or at most two drams of the salt in from a pint and a half to three pints of fluid, is the proper proportion; and this quantity may be taken at intervals in the course of the day, with the effect of powerfully increasing the activity of the kidneys, speedily putting an end to the acidulous state of the urine, and either washing out lithic grit and concretions of late formation from the pelvis of the kidney, from the ureter or bladder, or disintegrating and dissolving them, wherever they chance to be arrested. Should the urine not become neutral in the course of a day, the quantity of the alkali may be increased. That the soda may be thus taken for a great length of time with impunity, several remarkable cases prove, though we think that this should not lead to the indiscriminate exhibition and abuse of a valuable medicine. —*Medico-Chirurgical Review, July,* 1839.

